# Chimeric antigen receptor-T cells are effective against CEACAM5 expressing non-small cell lung cancer cells resistant to antibody-drug conjugates

**DOI:** 10.3389/fonc.2023.1124039

**Published:** 2023-02-27

**Authors:** Ye-Jin Kim, Wei Li, Doncho V. Zhelev, John W. Mellors, Dimiter S. Dimitrov, Du-San Baek

**Affiliations:** ^1^ Center for Antibody Therapeutics, Division of Infectious Diseases, Department of Medicine, University of Pittsburgh School of Medicine, Pittsburgh, PA, United States; ^2^ Abound Bio, Pittsburgh, PA, United States

**Keywords:** CEACAM5, NSCLCs, CAR-T, ADC, DM4 resistance, immunotherapy

## Abstract

Chimeric antigen receptor-T (CAR-T) cells and antibody-drug conjugates (ADCs) are promising therapeutic strategies in oncology. The carcinoembryonic antigen-related cell adhesion molecule 5 (CEACAM5) is overexpressed in tumors including non-small cell lung cancer (NSCLC) and pancreatic ductal adenocarcinoma (PDAC), and is an attractive target for therapies based on CAR-T cell or/and ADCs. We previously developed a highly specific antibody-based CAR-T cells targeting CEACAM5 and the tumoricidal effect of CAR-T cells was proved against neuro-endocrine prostate cancer (NEPC) cells expressing CEACAM5. Here, we compare the anti-tumor efficacy of our CAR-T cells with that of an anti-CEACAM5 ADC being clinically evaluated against NSCLC. Our anti-CEACAM5 CAR-T cells showed cytotoxicity in a CEACAM5 surface concentration dependent manner and reduced tumor growth in both ADC-responsive and -non-responsive CEACAM5-expressing NSCLC cells *in vitro* and *in vivo*. In contrast, the ADC exhibited cytotoxicity independent on the CEACAM5 cell surface concentration. Even though clinical translation of CEACAM5 targeting CAR-T cell therapies is still in preclinical stage, our CAR-T cell approach could provide a potential therapeutic strategy for CEACAM5-positive cancer patients with resistance to ADCs.

## 1 Introduction

Lung cancer is the prevalent cause of cancer related deaths worldwide (1). Recent advances in precision medicine have transformed lung cancer treatment from palliative chemotherapy to identification and targeting the genetic drivers of the disease (2, 3). Additionally, immunotherapy *via* the introduction of check point inhibitors has advanced further the methodologies for lung cancer treatment (4–6). The transformation of the way patients are treated has led to a significant prolongation of overall survival compared to traditional chemotherapy (5, 7) and to a continuous decrease in lung cancer-related deaths (8, 9). Non-small cell lung cancer (NSCLC) represents the most common and aggressive type of lung cancer (10) and development of drug resistance remains the main limitation for the treatment of NSCLC (5, 7, 11, 12). To address the challenge of drug resistance, new treatment approaches with a novel target are being sought. Recently, the human carcinoembryonic antigen-related cell adhesion molecule 5 (CEACAM5, CEA, or CD66e) has been identified as target for cancer immunotherapy (13).

CEACAM5 is a cell surface glycoprotein that is overexpressed in a variety of human tumors, including pancreatic cancers, breast cancers, lung cancer, and neuro-endocrine prostate cancer (NEPC) (14–16) and has been functionally associated with tumor differentiation, invasion, and metastasis (17–19). Currently, CEACAM5 has been targeted for developing immunotherapies such as bispecific T cell engagers (BiTEs), CAR-T cells, or ADCs (13, 16). The clinical trials using adoptive immunotherapies targeting CEACAM5 have shown limited success and have been complicated with adverse reactions. In contrast, the ADC, Tusamitamab Ravtansine (formerly SAR408701) has shown promising results and has been advanced to phase III trial (20, 21).

The anti-CEACAM5 ADC SAR408701, developed by Sanofi, is consisted of an anti-CEACAM5 antibody coupled to the maytansinoid agent DM4 with a cleavable linker, *N*-succinimidyl 4-(2-pyridyldithio) butyrate (SPDB) linker (16, 20). SAR408701 is administered intravenously as a conjugated antibody with an average drug-to-antibody ratio (DAR) of 3.8 (22). The binding of SAR408701 to CEACAM5 triggers antibody internalization, which eventually leads to release of the conjugated DM4 in a free form (23). The free DM4 binds to microtubules and suppresses their assembly (24), which leads to mitotic catastrophe (25). ADCs can potentially eliminate tumor cells by targeting tumor surface antigens acting as a membrane anchor. However, some ADCs show payload-induced toxicities, indicating limited therapeutic windows. In addition, oncogene mutations or resistance mechanisms can induce failure of ADCs (25–28). Possible resistance mechanisms of ADCs include: (1) less accessibility of ADC binding by reduced expression or mutation of target antigen (29), (2) high payload toxicity by up-regulation of drug efflux transporters (30), (3) changes in the intracellular routing or processing of ADCs (31), (4) payload drug resistance by tumor heterogeneity (32), and other mechanisms (25, 33). One approach for treating tumors exhibiting resistance to ADCs is to use CAR-T cells or BiTEs targeting the same antigen as the ADCs. CAR-T cell therapy is composed of T cells collected from autologous peripheral blood and engineered to express CARs specifically directing against the tumor surface antigen of interest (34). We previously identified a novel, fully human monoclonal antibody, 1G9, targeting membrane-proximal region of CEACAM5. CAR-T cells guided by the scFv 1G9 exhibited a potent cytotoxicity for NEPC *in vitro* and *in vivo* (35).

Here, we designed a model system using cell lines derived from NSCLC tumors, which are resistant to DM4 or an in-house developed analog of SAR408701, and our anti-CEACAM5 CAR-T cells showed CEACAM5-specific anti-tumor activities for DM4-resistant NSCLCs *in vitro* and *in vivo*. Our results suggest the potential of CAR-T cells-based approaches as a therapeutic strategy for ADCs-non-responsive patients.

## 2 Materials and methods

### 2.1 Cell lines

H1975, A549, H1299, H2030, H2009, HPAC and HPAF-II cells were purchased from ATCC. H1975, A549, H1299, H2030, and H2009 cells were maintained in RPMI1640 (Gibco) supplemented with 10% v/v FBS (Gibco) and 1% penicillin-streptomycin (P/S, Gibco). HPAC cells were cultured in F12K (ATCC) with 10% FBS and 1% P/S. HPAF-II cells were maintained in EMEM (ATCC) with 10% FBS and 1% P/S. H1975-CEACAM5, H2009-CEACAM5, A549-CEACAM5, and H1299-CEACAM5, stably expressing CEACAM5, were generated by stable infection with lentivirus from the CEACAM5 lentiviral plasmid (Origene) using a commonly used protocol (36). Stably transfected cells were selected in RPMI1640 supplemented with 10% FBS, 1% P/S and 1 μg/ml (for A549-CEACAM5) or 2 μg/ml (for H1975-CEACAM5, H2009-CEACAM5, and H1299-CEACAM5) puromycin (Gibco). The cell surface CEACAM5 expression of the stably transfected cells was then assessed with PE-conjugated anti-CEACAM5 IgG1 (Miltenyi Biotec, 130-114-217) by flow cytometry. Anti-CEACAM5 CAR-T cells were generated as previously described (35) and expanded in the T cell media (RPMI1640 supplemented with extra 2mM glutamine, 10% human serum, and 1% P/S) in the presence of hIL-2 (fed every 2 days, 50 IU/ml, Miltenyi Biotec).

### 2.2 Tissue microarray and immunohistochemistry

Human tumor and normal multiple frozen tissue arrays were purchased from Fisher scientific (50-180-886). 14 tumors and 14 correspondent normal tissues (brain, breast, colon, muscle, kidney, liver, lung, pancreas, prostate, skin, small intestine, stomach, ovary, and uterus) were mounted on a positively charged glass slide. For immunohistochemistry, the tissue slide was blocked with 10% normal horse serum for 1 h at 25°C and incubated with mouse anti-human CEACAM5 antibody (Novus biologicals, NB11058734, 1:100) for overnight at 4°C in humidified chamber. The tissue slide was washed with PBS, incubated with biotin-conjugated anti-mouse antibody (Invitrogen, B2763, 1:200) for 1 h at 25°C, and washed again with PBS. Slide was incubated with ImmunoCruz ABC staining (Santacruz, sc-516216) by the manufacturer instructions. Stains were then visualized using DAB peroxidase substrate (Santacruz, sc-249982). The positive pixel areas of CEACAM5 staining of the entire tissue were quantified using Image J software and the total CEACAM5 area of tumor tissue normalized to the total CEACAM5 pixel area of the corresponding normal tissue.

### 2.3 *In vitro* cell cytotoxicity assay

The cell killing activity of DM4 or the ADC SAR408701 analog was measured by CellTiter-Glo Luminescent cell viability assay kit (Promega, G7571). Cells (2.5×10^3^ cells/well in white 96-well plate) were cultured for 12 h prior to treatment with the indicated doses of DM4 or ADC for 96 h at 37°C. Normalized % ATP values were calculated by normalizing luminescence values for buffer (DPBS or DMSO)-treated cells. The LDH-Glo cytotoxicity assay kit (Promega, J2381) was used to measure cell viability in treatment of anti-CEACAM5 CAR-T cells. Control T or CAR-T cells as effector cells were incubated with target cells (5×10^3^ cells/well in 96-well plate) at the indicated E:T ratio for 24 h at 37°C. Controls conducted for the calculation of percent cytotoxicity were included according to the manufacturer’s instructions.

### 2.4 *In vivo* study

All studies were approved by the University of Pittsburgh institutional Animal Care and Use Committee. A549 cells (7×10^6^/mice), A549-CEACAM5 cells (7×10^6^/mice) or H1975-CEACAM5 cells (5×10^6^/mice) resuspended in 200 μl of DPBS were subcutaneously injected into the right flank of female NSG mice (6-8 weeks old, The Jackson Laboratory). When the tumor volume reached approximately 150 mm^3^, mice were intravenously treated with ADC SAR408701 analog (10 mg/kg or 5 mg/kg), Control T (5x10^6^/mice) or anti-CEACAM5 CAR-T cells (5×10^6^ or 2×10^6^/mice) every 4 days, two times, *via* tail vein. Tumor volume was measured by two-dimensional measurements with a caliper and calculated according to the formula V=0.5 × length × (width)^2^. Tumor growth inhibition (TGI) by anti-CEACAM5 ADC or CAR-T compared to that by vehicle or control T was determined on the last day of the study according to the formula: TGI (%) = [1-( ${\text{V}}_{f}^{\mathrm{treated}}$ - ${\text{V}}_{i}^{\mathrm{treated}}$ )/( ${\text{V}}_{f}^{\mathrm{control}}$ - ${\text{V}}_{i}^{\mathrm{control}}$ )] ×100, where V_f_ is the final mean tumor volume in the treated group (ADC or CAR-T cells), and V_i_ is the initial mean tumor volume in the control group (vehicle or control T cells). Animals were euthanized when the tumor volume reached >1-1.5 cm^3^.

### 2.5 Statistical analysis

Statistical analyses were performed using GraphPad Prism software (GraphPad, Inc.). Data are presented as the mean ±SD for representative data from three independent experiments. One-way analysis of variance (ANOVA) with Tukey’s *post hoc* test was used to evaluate the significance of differences. Survival curve was represented as Kaplan-Meier plots, with statistical significance determined by log-rank (Mantel-Cox) tests. *P* values less than 0.05 were considered statistically significant. *P* values less than 0.05, 0.01, 0.001, and 0.0001 are indicated as *, **, ***, and ****, in the respective figure.

## 3 Results

### 3.1 CEACAM5 protein expression in human tumor specimens and normal tissues

**Figure 1:**
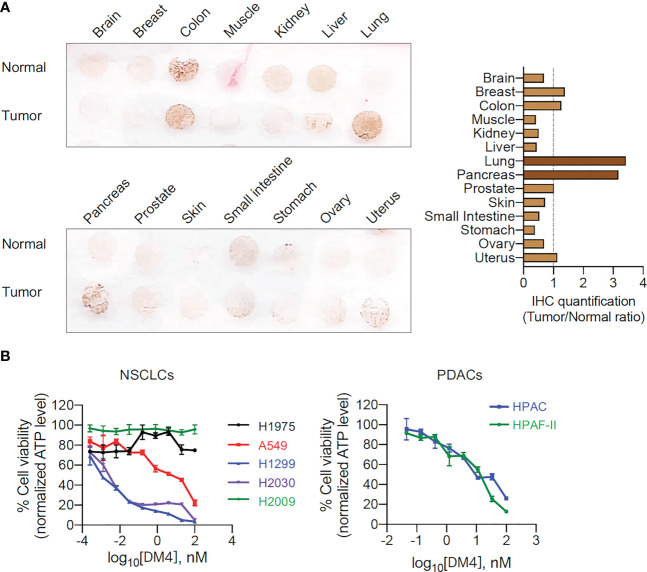
CEACAM5 expressions in different normal/tumor tissues and DM4 sensitivity in NSCLCs and PDACs. **(A)** CEACAM5 immunohistochemistry (IHC) of 14 tumor tissues and 14 correspondent normal tissues in tissue microarray (left panel) and quantification values of IHC images (right panel). **(B)** DM4 response curves in NSCLCs (H1975, A549, H1299, H2030, and H2009 cells) and PDACs (HPAC and HPAF-II cells). Normalized % cell viability (ATP level) was calculated by normalizing luminescence values using buffer (DMSO)-treated respective cells. Results are shown as the mean ±SD for representative data from three independent experiments.

To verify protein expression of CEACAM5 in human tissues, we performed IHC analysis with 14 different human tumor and 14 correspondent normal tissues (brain, breast, colon, muscle, kidney, liver, lung, pancreas, prostate, skin, small intestine, stomach, ovary, and uterus) on the tissue microarray. Lung tumor and pancreas tumor highly expressed CEACAM5 compared to normal lung and pancreas tissues (**Figure 1A**). In contrast, CEACAM5 expression was detected in normal tissue as well as tumor tissue from the colon, and no CEACAM5 was observed in normal and tumor of other tissues, as reported previously (16, 37). Recent data indicated that prostate cancer subtypes are differentiated as prostate adenocarcinoma (PrAd) and neuroendocrine prostate cancer (NEPC) (37). CEACAM5 is especially prominent as a therapeutic target in NEPC (35). In accordance with CEACAM5 overexpression in lung tumor tissues, the anti-CEACAM5 antibody drug-conjugate (ADC) SAR408701 is being evaluated in non-small cell lung cancer (NSCLC) with different interventions in clinical trials (16, 20).

### 3.2 DM4 sensitivity in NSCLCs and PDACs

DM4 is a potent cytotoxic agent derived from maytansine that blocks tubulin polymerization and is used as a payload for ADC SAR408701. We determined the sensitivity of NSCLC cells (NSCLCs) and PDAC cells (PDACs) to DM4. Three NSCLCs, A549, H2030, and H1299 cells, responded to treatment with DM4, but two NSCLCs, H1975 and H2009 cells, showed resistance to DM4 (**Figure 1B**). The two tested PDACs, HPAC and HPAF-II cells, were sensitive to DM4 *in vitro* (**Figure 1B**). Vecchione, L. et al. reported that BRAF(V600E) is a predictive biomarker of the DM4 response in colon cancer PDX models (26). To identify whether the DM4 sensitivity would be predicted by oncogene mutation status, we examined oncogene mutations through Cancer Cell Line Encyclopedia (CCLE) database (38). No correlation was found between the observed DM4 sensitivity and oncogene(s) mutation status (**Supplementary Table S1**).

### 3.3 *In vitro* effects of CAR-T cells and ADC targeting CEACAM5 in DM4^S^ and DM4^R^ cell lines

**Figure 2:**
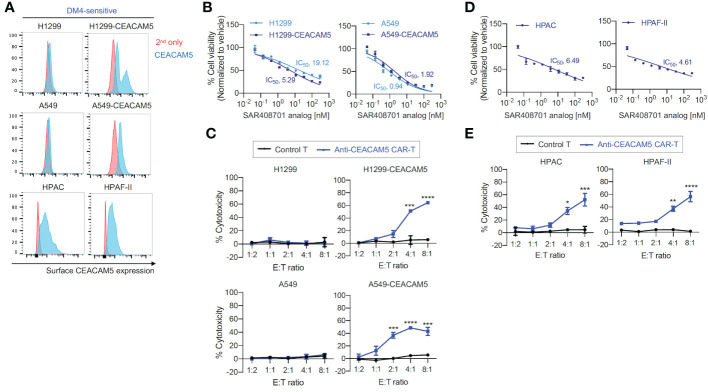
*in vitro* cell killing activities of the ADC SAR408701 analog and CAR-T cells targeting CEACAM5 in DM4^S^ cells. **(A)** Cell surface CEACAM5 expression levels in DM4^S^ NSCLCs (H1299, H1299-CEACAM5, A549, and A549-CEACAM5 cells) and PDACs (HPAC and HPAF-II cells). **(B, D)** Cell killing activities with ADC SAR408701 analog against DM4^S^ NSCLCs (H1299, H1299-CEACAM5, and A549, A549-CEACAM5 cells) **(B)** and DM4^S^ PDACs (HPAC, and HPAF-II cells) **(D)**. Normalized % cell viability (ATP level) was calculated by normalizing luminescence values using vehicle (buffer)-treated respective cells. The IC_50_ were then determined by nonlinear regression plot of percent specific cytotoxicity versus Log10 concentration of ADC SAR408701 analog using GraphPad Prism software. **(C, E)** Cytotoxic activities (%) of anti-CEACAM5 CAR-T cells against DM4^S^ NSCLCs **(C)** and PDACs **(E)**. Significance was tested using one-way ANOVA, followed by the tukey’s multiple *post hoc* test. ****, *P*<0.0001; ***, *P*<0.001; **, *P*<0.01; *, *P*<0.05; versus control T at each E:T ratio. **(B–E)** Results are shown as the mean ±SD for representative data from three independent experiments.

To test *in vitro* cytotoxicity of CAR-T cells and ADC targeting CEACAM5, we successfully produced an analog of ADC SAR408701 derived from Sanofi (16). The ADC SAR408701 analog, produced by NJ Biopharmaceuticals, has a DAR of 3.7 as determined by reverse-phase (RP) chromatography coupled with mass spectrum (MS) analysis, and exhibits a homogenous folding as tested by size-exclusion chromatography (SEC) (**Supplementary Figure S1**). The CEACAM5 binding specificity and affinity of in-house developed ADC SAR408701 analog were also confirmed (data not shown). The CEACAM5 surface expression was first screened in 7 lung cancer cell lines, 4 pancreatic cancer cell lines, and 3 other cancer cell lines (**Supplementary Figure S2**). Unfortunately, total 7 lung cancer cell lines exhibited no CEACAM5 surface expression, so we generated CEACAM5-stably expressing DM4-sensitive (DM4^S^) NSCLCs, H1299-CEACAM5 and A549- CEACAM5. CEACAM5 expression was confirmed by flow cytometry (**Figure 2A**). ADC SAR408701 analog was tested in dose-response cell viability assays using four DM4^S^ CEACAM5-positive cell lines (H1299-CEACAM5, A549-CEACAM5, HPAC, and HPAF-II) and two DM4^S^ CEACAM5-negative cell lines (H1299 and A549). Treatment with ADC SAR408701 analog reduced cell viability in a dose-dependent manner with all CEACAM5-positive cells (**Figures 2B, D**). However, the ADC SAR408701 analog also showed a killing activity in DM4^S^ CEACAM5-negative H1299 and A549 cell lines (**Figure 2B**) which may be attributed to the payload-induced toxicity independent of direct antigen-mediated internalization (39, 40). We next assessed the cytotoxicity of our anti-CEACAM5 CAR-T cells for DM4^S^ NSCLCs and PDACs *in vitro.* Our anti-CEACAM5 CAR-T cells were previously generated and evaluated in prostate cancer cells *in vitro* and *in vivo* (35). Anti-CEACAM5 CAR-T cells showed a potent cytotoxicity against CEACAM5-positive NSCLCs (H1299-CEACAM5 and A549-CEACAM5) (**Figure 2C**) and PDACs (HPAC and HPAF-II) (**Figure 2E**). Contrary to the ADC SAR408701 analog, CAR-T cells did not exhibit non-specific toxicity in CEACAM5-negative NSCLCs (H1299 and A549) (**Figure 2C**).

**Figure 3:**
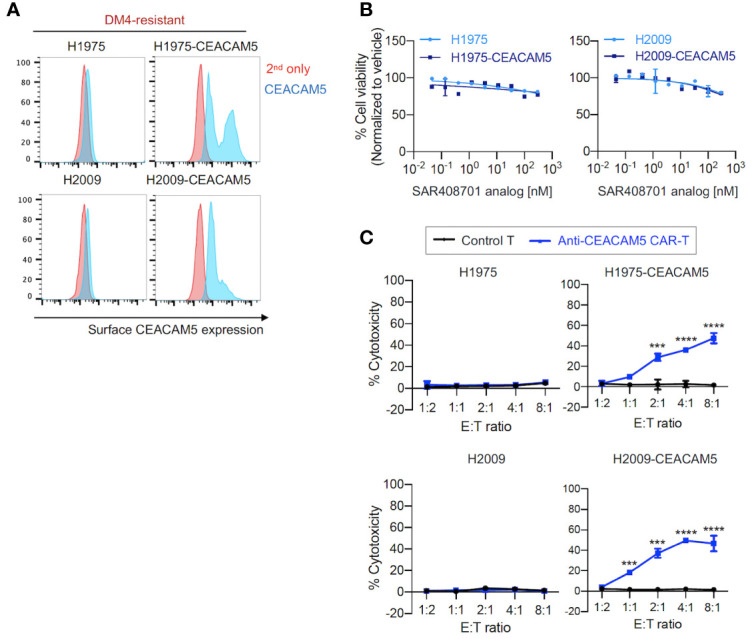
*in vitro* cell cytotoxicity assay of the ADC SAR408701 analog and CAR-T cells targeting CEACAM5 in DM4^R^ cells. **(A)** Cell surface CEACAM5 expression levels in DM4^R^ NSCLCs (H1975, H1975-CEACAM5, H2009, and H2009-CEACAM5 cells). **(B)** Cell killing activities with ADC SAR408701 analog against CEACAM5-positive DM4^R^ cell lines (H1975-CEACAM5 and H2009-CEACAM5 cells) and CEACAM5-negative DM4^R^ cell lines (H1975 and H2009 cells). Normalized % cell viability (ATP level) was calculated by normalizing luminescence values for vehicle (buffer)-treated respective cells. **(C)** Cytotoxic activities (%) of anti-CEACAM5 CAR-T cells against CEACAM5-positive and CEACAM5-negative DM4^R^ NSCLC cells. Significance was tested using one-way ANOVA, followed by the tukey’s multiple *post hoc* test. ****, *P*<0.0001; ***, *P*<0.001; versus control T at each E:T ratio. **(B, C)** Results are shown as the mean ±SD for representative data from three independent experiments.

Second, we evaluated the effects of anti-CEACAM5 ADC SAR408701 analog and CAR-T cells on DM4-resistant (DM4^R^) NSCLCs (H1975 and H2009). CEACAM5-expressing NSCLCs - H1975-CEACAM5 and H2009-CEACAM5, were constructed (**Figure 3A**). The DM4^R^ NSCLCs expressing or not expressing CEACAM5 showed a mild concentration-dependent response to treatment with the ADC SAR408701 analog (measured maximum of 20% cytotoxicity) (**Figure 3B**). In contrast, the anti-CEACAM5 CAR-T cells demonstrated a strong E:T ratio-dependent cytotoxicity for cells expressing CEACAM5 and no response to cells not expressing CEACAM5 (**Figure 3C**).

### 3.4 *In vivo* tumor growth inhibition by CAR-T cells and ADC targeting CEACAM5 in DM4^S^ and DM4^R^ NSCLCs tumors

**Figure 4:**
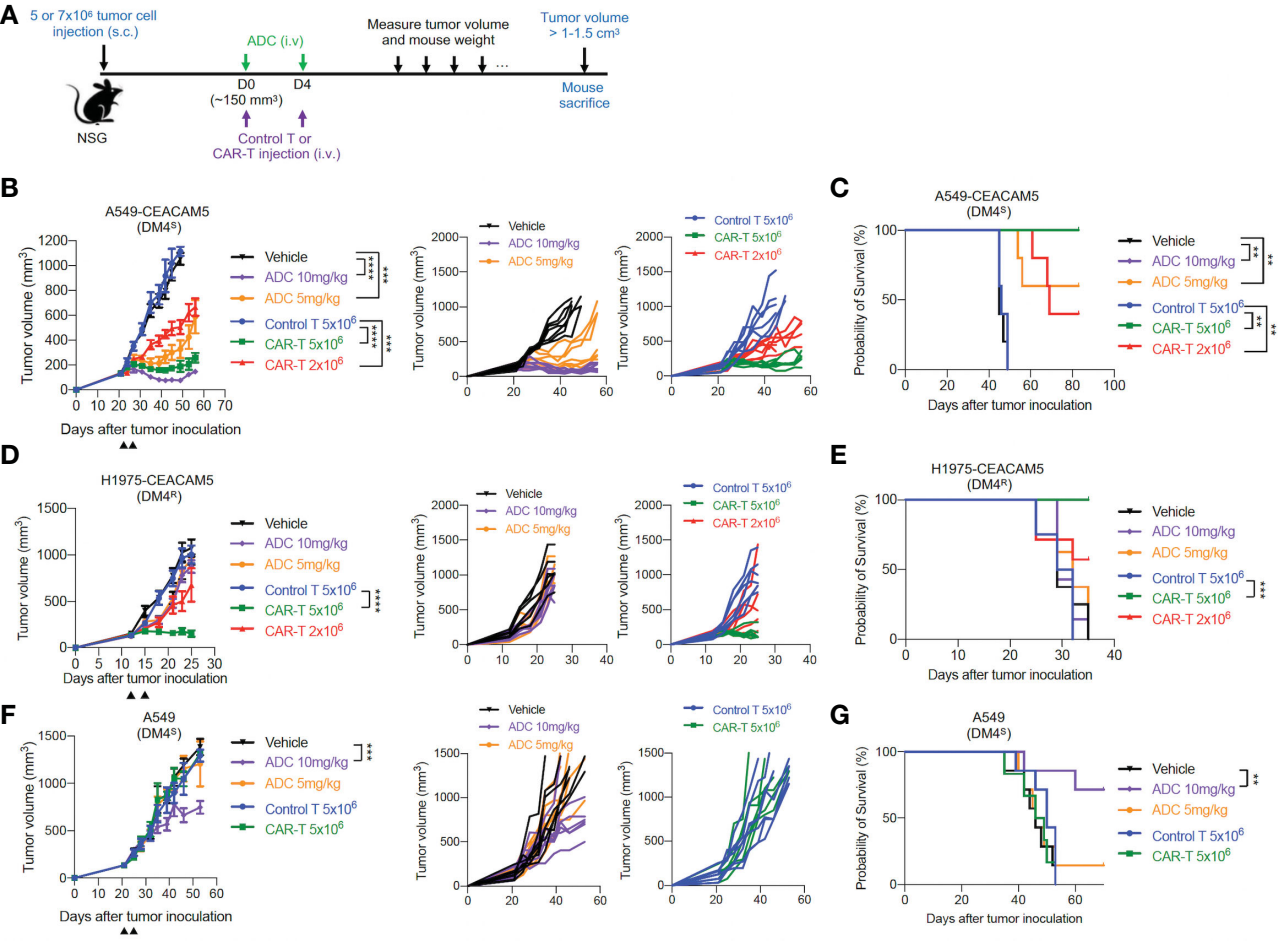
*in vivo* anti-tumor activities of the ADC SAR408701 analog and CAR-T cells targeting CEACAM5 in DM4^S^ and DM4^R^ NSCLC tumors. **(A)** Schematic representation of experimental design and treatment schedule for mice studies. **(B, D, F)** Tumor growth curve (left panels) and individual mice curves (right panels) of DM4^S^ A549-CEACAM5 **(B)**, DM4^R^ H1975-CEACAM5 **(D)**, and CEACAM5-negative DM4^S^ A549 **(F)** tumors. Significance was analyzed by comparing the tumor volume (mm^3^) ±SD at endpoint of 1.0 cm^3^ **(B)** or end day (**D, F**) and determined using one-way ANOVA, followed by the tukey’s multiple *post hoc* test. ****, *P*<0.0001; ***, *P*<0.001. **(C, E, G)** Survival curve showing the efficacy of the ADC SAR408701 analog and CAR-T cells in NSG mice. Tumor volume (mm^3^) are shown as mean ±SD for n=6 or 7 per group. Survival was presented by Kaplan-Meier plot of percentage of mice with tumor volume ≥ 1-1.5 cm^3^. Significance was determined by log-rank (Mantel-Cox) test. ***, *P*<0.001; **, *P*<0.01.

To further examine the therapeutic potentials of our CAR-T cells *in vivo*, we utilized mouse xenograft tumor models of CEACAM5-expressing DM4^S^ (A549-CEACAM5), DM4^R^ (H1975-CEACAM5) and CEACAM5-negative DM4^S^ (A549) NSCLCs. Anti-CEACAM5 CAR-T cells and ADC SAR408701 were administered as **Figure 4A**. In mice bearing DM4^S^ A549-CEACAM5, ADC SAR408701 analog treatment significantly reduced tumor growth and improved mouse survival in dose-dependent manner. CAR-T cells also showed a dose-dependent tumor growth inhibition (**Figure 4B**) and extended survival in CEACAM5-positive DM4^S^ A549-CEACAM5 (**Figure 4C**). However, a different result was observed in the DM4^R^ H1975-CEACAM5 tumors, where potent anti-tumor activity was observed for CAR-T cells-treated group, but not for the ADC SAR408701 analog treated group (**Figures 4D, E**). We sought further insights into the tumor growth inhibition activity of anti-CEACAM5 CAR-T and ADC SAR408701 analog by examining their activity in a CEACAM5-negative DM4^S^ A549 tumor model, because the ADC SAR408701 analog showed a CEACAM5-independent killing activity *in vitro* cell-based system. The high dose (10 mg/kg) of ADC SAR408701 analog suppressed A549 tumor growth (51% TGI) even in the absence of expressed CEACAM5, but anti-CEACAM5 CAR-T displayed no tumor growth inhibition (**Figures 4F, G**). The mouse body weight, monitored as an indicator of drug toxicity, was similar compared with vehicle group (**Supplementary Figure S3**). These results indicate that our CAR-T cells therapy is effective and safe against NSCLCs and can be an alternative treatment strategy in ADC-non-responsive NSCLCs.

## 4 Discussion

NSCLC is a primary type of lung cancer and one of the most common malignant tumors on a global scale (9, 41). CEACAM5, a glycosylated transmembrane protein, is often presented in lung cancer tumor tissues (42). CEACAM5-targeted therapies, including CAR-T cells (43) or ADCs (20), have been developed against lung cancer. Here, we investigated the efficacy of such therapeutic modalities targeting CEACAM5 in ADC-sensitive and -resistant NSCLC cell lines. CAR-T cells mediated MHC-unrestricted tumor cell killing by enabling T cells to bind target cell surface antigens (44). CAR-T cell therapies are being developed as potentially powerful immunotherapeutic tools. However, they remain unable successfully fight solid tumors in their current state (45, 46) due to heterogeneous tumor antigen expression (47), the immunosuppressive tumor microenvironment (48) and T cell exhaustion driven by chronic antigen exposure (49). Another targeted approach, ADC, is an evolving class of immunotherapeutics that consist of a cytotoxic agents linked covalently to an antibody. ADCs act through a series of processes including target cell binding, internalization, and release of cytotoxic payload (50). ADCs can potentially eliminate tumor cells by targeting tumor surface antigens. In some cases, however, neighboring cancer cells (bystander effects) or normal cells (toxicity) that do not express the tumor surface antigen can be abolished (51).

The preclinically validated anti-CEACAM5 ADC, SAR408701, was developed by Sanofi, and it is comprised of the antibody SAR408377 covalently linked to the cytotoxic agent maytansinoid DM4, a potent microtubule-destabilizing agent (16). SAR408701 is currently being evaluated in advanced colorectal, gastric, and non-small cell lung cancer patients. In an interim analysis of a first in-human study (NCT02187848) in patients with non-squamous NSCLC, SAR408701 showed an objective response rate (ORR) of only 23%, even in patients with ≥ 50% of CEACAM5-expressing tumor cells. One possibility for this significant difference could be the emergence of ADC resistant subclones caused by cell surface recycling of the targeted tumor antigen, altered internalization, or impaired release of the toxic payload into the cytosol (29, 52). In this study, we examined the response of NSCLCs or PDACs to single drug DM4, the payload of SAR408701, and identified that the ADC response of cell lines used in this study was determined by payload DM4 response. We compared the cytotoxic effects of in-house developed ADC SAR408701 analog and our anti-CEACAM5 CAR-T (35) to DM4^S^ and DM4^R^ NSCLCs and PDACs. Our anti-CEACAM5 CAR-T cells exhibited a potent cytotoxicity for both DM4^S^ and DM4^R^ CEACAM5-expressing NSCLCs or PDACs, both *in vitro* and *in vivo*. By contrast, SAR408701 analog only showed cytotoxicity to DM4^S^ cells. Also, the ADC SAR408701 analog exhibited killing effects against DM4^S^ NSCLCs irrespective of CEACAM5 expression. CEACAM5 was previously reported as a non-internalizing receptor or very slow internalization receptor (53). This property of CEACAM5 may contribute to the different efficacy of CAR-T cells and ADCs, but further experimentation to examine the detailed mechanism is needed.

In this study, two promising therapeutic strategies in oncology, CAR-T cell therapy and ADC, are compared in terms of efficacy and toxicity. Both strategies represent promising therapeutic modalities in spite of many safety issues in trials (54), lack of transparency in data sharing (55). Accordingly customized patient selection for each therapy is important (56, 57). In this regard, our anti-CEACAM5 CAR-T cells therapy can be a promising candidate for development as a potential treatment for ADC-non-responsive patients with CEACAM5-positive tumors.

## Data availability statement

The original contributions presented in the study are included in the article/**Supplementary Material**. Further inquiries can be directed to the corresponding authors.

## Ethics statement

The animal study was reviewed and approved by University of Pittsburgh institutional Animal Care and Use Committee.

## Author contributions

Y-JK, JM, DD, and D-SB wrote the manuscript and analyzed the data. Y-JK and D-SB designed and performed the experiments. WL, DZ, and JM interpreted the data and edited the manuscript. All authors contributed to the article and approved the submitted version.
