# Supplementary material for: Chimeric antigen receptor-T cells are effective against CEACAM5 expressing non-small cell lung cancer cells resistant to antibody-drug conjugates
Source: Front Oncol. 2023 Feb 27;13:1124039. doi: 10.3389/fonc.2023.1124039 (PMC10010383; doi:10.3389/fonc.2023.1124039)
Supplement: Supplementary file 1 [file DataSheet_1.docx]

Supplementary Material

# Supplementary Figures and Table

## Supplementary Table

Oncogenes mutations status and DM4 response of NSCLCs and PDACs used in this study.

## Supplementary Figures


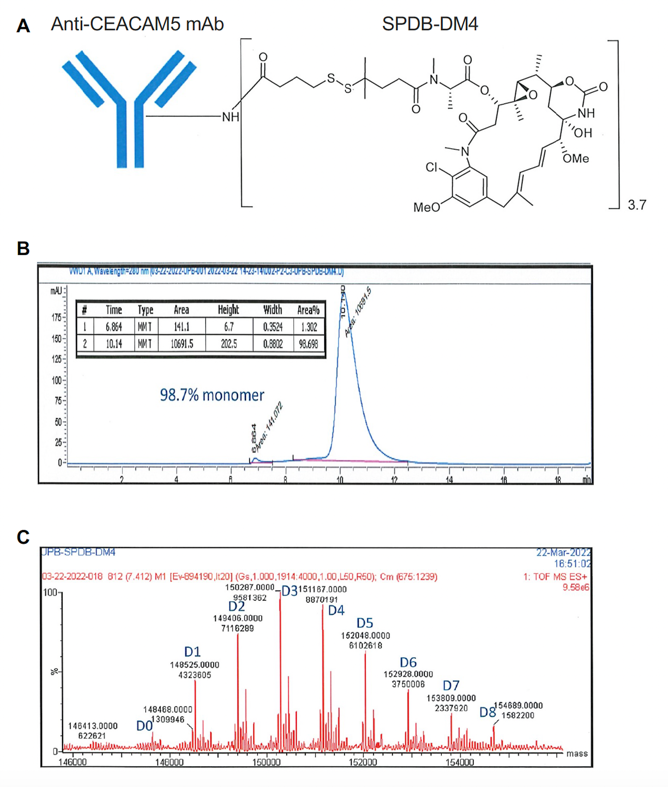


**Supplementary Figure S1. (A)** Schematic structure of ADC SAR408701 analog, anti-CEACAM5 antibody covalently linked to the cytotoxic agent DM4 via an N-succinimidyl 4-(2-pyridyldithio) butyrate (SPDB) linker. **(B)** Monomer determination by SEC (15 μg of ADC injected over TSKgelG3000SWXL). **(C)** Drug-to-antibody ratio (DAR), 3.7, determination by RP-MS analysis (15 μg of ADC injected over PLRP-S).

**
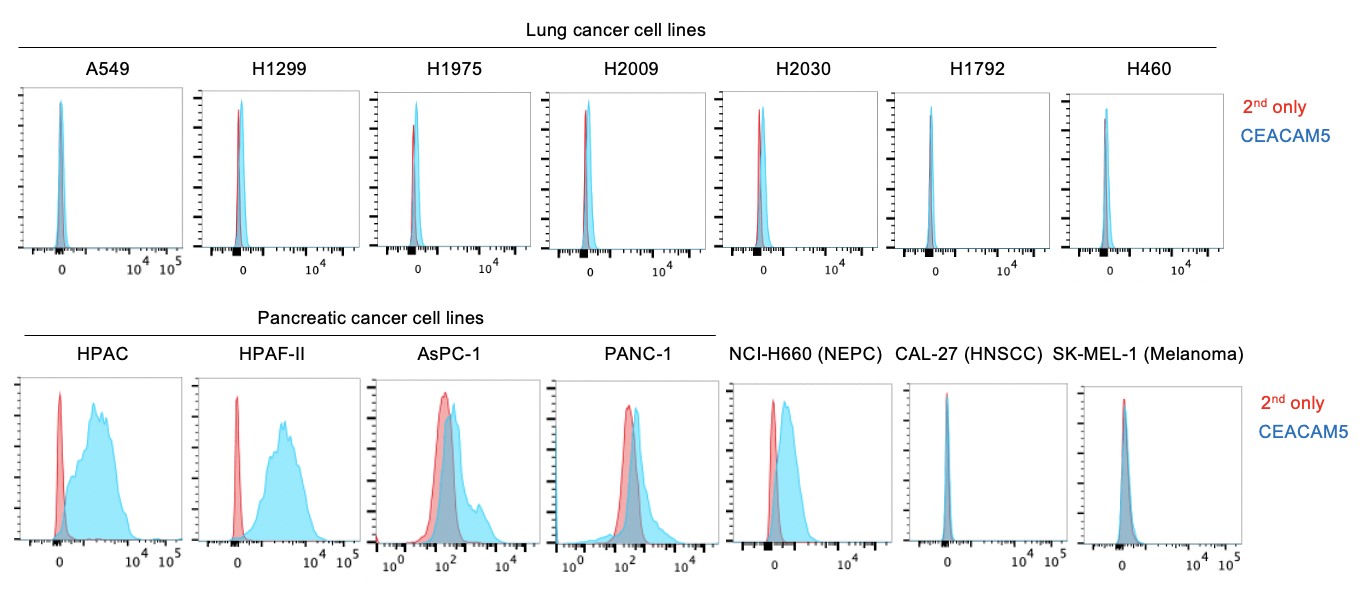
**

**Supplementary Figure S2.** Cell surface expression level of CEACAM5 on lung cancer cells, pancreatic cancer cells, neuro-endocrine prostate cancer (NEPC) cells, head and neck squamous cell carcinoma (HNSCC), and melanoma cells.


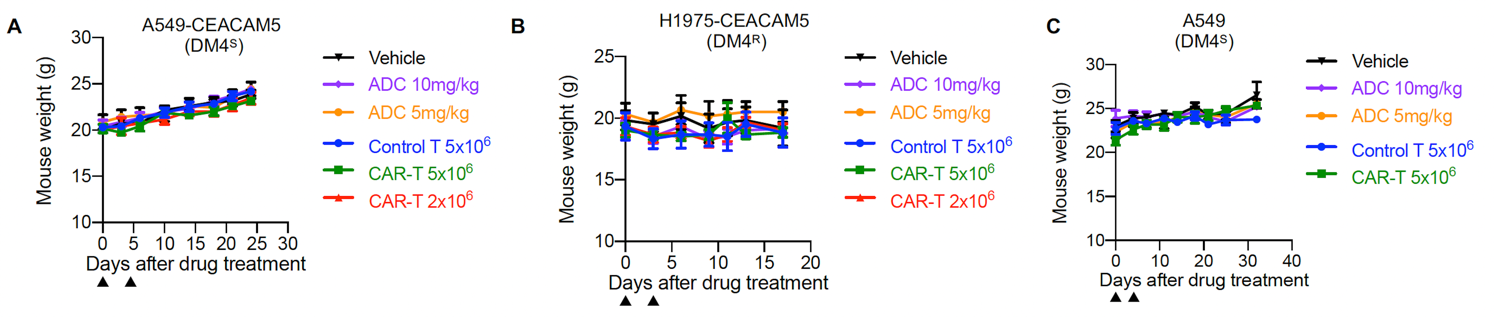


**Supplementary Figure S3. (A, B and C)** Mouse weight (g) ± SD versus time (days) from mice (n=6 or 7) injected with cells (**A**, DM4^S^ A549-CEACAM5, **B**, DM4^R^ H1975-CEACAM5, or **C**, CEACAM5-negative DM4^S^ A549) and treated with the ADC SAR408701 analog and CAR-T cells until tumor volume 1-1.5 cm^3^, as described in Figure 4.
